# Challenges for cancer patients returning home during SARS-COV-19 pandemic after medical tourism - a consensus report by the emirates oncology task force

**DOI:** 10.1186/s12885-020-07115-6

**Published:** 2020-07-10

**Authors:** Humaid O. Al-Shamsi, Ibrahim Abu-Gheida, Shabeeha K. Rana, Neil Nijhawan, Ahmed S. Abdulsamad, Sadir Alrawi, Mohamed Abuhaleeqa, Taleb M. Almansoori, Thamir Alkasab, Essa M. Aleassa, Martine C. McManus

**Affiliations:** 1 College of Medicine, University of Sharjah, Sharjah, United Arab Emirates; 2 Emirates Oncology Task Force, Emirates Oncology Society, Dubai, United Arab Emirates; 3 Department of Oncology - Alzahra Hospital – Dubai, United Arab Emirates and Department of Medicine, University of Sharjah, Sharjah, United Arab Emirates; 4 Burjeel Medical City, Abu-Dhabi, United Arab Emirates; 5 College of Medicine and Health Sciences, United Arab Emirates University, Al Ain, United Arab Emirates; 6 Sheikh Khalifa Medical City, Abu Dhabi, United Arab Emirates; 7 Radiology Department, College of Medicine and Health Sciences, UAE University, Abu Dhabi, United Arab Emirates; 8 Section of Hepato-Pancreato-Biliary Surgery, Department of General Surgery, Digestive Disease and Surgery Institute, Cleveland Clinic Foundation, Cleveland, OH USA; 9 Department of Surgery, College of Medicine and Health Sciences, United Arab Emirates University, Al Ain, United Arab Emirates

## Abstract

**Background:**

The COVID-19 pandemic has caused a global health crisis. Numerous cancer patients from non-Western countries, including the United Arab Emirates (UAE), seek cancer care outside their home countries and many are sponsored by their governments for treatment. Many patients interrupted their cancer treatment abruptly and so returned to their home countries with unique challenges. In this review we will discuss practical challenges and recommendations for all cancer patients returning to their home countries from treatment abroad.

**Method:**

Experts from medical, surgical and other cancer subspecialties in the UAE were invited to form a taskforce to address challenges and propose recommendations for patients returning home from abroad after medical tourism during the SARS-COV-19 Pandemic.

**Results:**

The taskforce which consisted of experts from medical oncology, hematology, surgical oncology, radiation oncology, pathology, radiology and palliative care summarized the current challenges and suggested a practical approaches to address these specific challenges to improve the returning cancer patients care.

Lack of medical documentation, pathology specimens and radiology images are one of the major limitations on the continuation of the cancer care for returning patients. Difference in approaches and treatment recommendations between the existing treating oncologists abroad and receiving oncologists in the UAE regarding the optimal management which can be addressed by early and empathic communications with patients and by engaging the previous treating oncologists in treatment planning based on the available resources and expertise in the UAE. Interruption of curative radiotherapy (RT) schedules which can potentially increase risk of treatment failure has been a major challenge, RT dose-compensation calculation should be considered in these circumstances.

**Conclusion:**

The importance of a thorough clinical handover cannot be overstated and regulatory bodies are needed to prevent what can be considered unethical procedure towards returning cancer patients with lack of an effective handover. Clear communication is paramount to gain the trust of returning patients and their families. This pandemic may also serve as an opportunity to encourage patients to receive treatment locally in their home country. Future studies will be needed to address the steps to retain cancer patients in the UAE rather than seeking cancer treatment abroad.

## Background

The emergence of coronavirus disease 2019 (COVID-19) has caused a global public health emergency. In December 2019, an outbreak of respiratory disease caused by a novel coronavirus was first detected in China, and has now spread to more than 200 countries [1, 2]. The virus was named Severe Acute Respiratory Syndrome Coronavirus 2, “SARS-CoV-2”, and has a phylogenetic similarity to SARS-CoV-2 that caused the SARS pandemic in 2002 [3]. This new type of respiratory illness is characterized by rapid human-to-human transmission having achieved pandemic spread [4]. There are currently no vaccines available and there is presumably no pre-existing immunity in the population. In the UAE, as of June 16th 2020, there were 42.982 confirmed COVID-19 cases and 293 COVID-19 related mortalities [5].

Medical tourism is defined as the process of intentional travel outside the country of residence for the purpose of receiving medical care [6]. Patients with cancer, living in developing countries, are attracted by specialized care such as sophisticated surgery or the latest advancements in Anti-Cancer therapy offered in developed nations. However, numerous problems may arise with complications, including misunderstandings caused by language differences, cultural discrepancies, ethical and religious issues, lack of adequate information, and delayed arrival as well as risks associated with travel, extensive medical expenses and issues with patient insurance [7].

In the UAE where cancer is the third leading cause of death, a significant number of patients receive their cancer care and follow up outside the UAE [8]. However, it is important to note that due to the current global pandemic, cancer patients in the UAE are unable to travel abroad for treatment due to the country wide lockdown. The UAE government has suspended all funding for offshore medical treatment, including oncology care, to avoid placing patients and companions at a higher risk of acquiring the virus. This suspension applies to patients already undergoing cancer treatment overseas and patients newly diagnosed with malignancies requiring treatment now readily available abroad [9]. Therefore, cancer patients in the UAE are experiencing challenges as the current travel restrictions prevent them seeking health services abroad.

Moreover, in the UAE, there are significant misconceptions about the quality of the health and cancer care available. For example, the health system in the UAE was ranked 27th in the world by the World Health Organization (WHO) in 2010 [10]. As a result, many patients, and largely their families, demand second opinions for cancer treatment despite the extensive oncological treatment options being available in the UAE [9]. However, because of a lack of official data in this regard, it is difficult to quantify the percentage of patients whose cancer treatment can, in fact, only be managed abroad. We estimate that less than 10% of patients truly require specialized care abroad, including access to clinical trials. Consequently, medical tourism results largely from the patients’ and their families’ belief that newer technologies and better medicine exist overseas, and therefore, patients’ outcomes will be improved [9, 11].

This consensus report by the Emirates Oncology Task Force discusses practical challenges and recommendations for those cancer patients returning to the UAE from treatment abroad, although it is also applicable to other patients from different parts of the world seeking medical tourism. The aim of this report is to provide recommendations and guidance for treating oncologists to manage these unique clinical challenges.

**Fig. 1:**
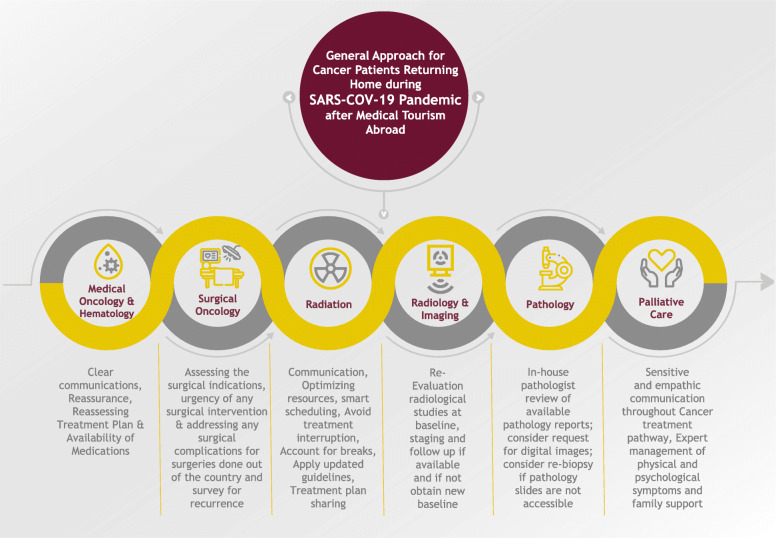
An outline for the challenges & recommendations for cancer patients returning from treatment abroad after medical tourism during the COVID-19 pandemic

An outline for the Challenges & Recommendations for Cancer Patients Returning from Treatment Abroad During the COVID-19 Pandemic, Fig. 1.

### Medical oncology

One of the major challenges is the quarantine period that cancer patients face upon return to the UAE, which causes significant issues with this vulnerable population. The quarantine is part of the UAE’s strategy to mitigate the spread of COVID-19 from which travelers are released after 14 days and following 2 negative nasopharyngeal swab Polymerase Chain Reaction (PCR) tests. One of the many challenges includes accessing specialized oncology care for the management of pain control, surgical complications and infectious complications of cancer treatment.

Other important challenges include missing documentation, such as treatment plans and chemotherapy protocols from previous oncology providers. This can be addressed by getting access to the electronic medical records from the patients’ portals, yet often patients do not have access to them. Communication with the previous treating providers can also be challenging with slow responses to requests for medical records which can delay the treatment plans for these patients. Where medical records are neither readily available nor accessible, baseline staging using biopsy, imaging and multidisciplinary team (MDT) evaluation is the most appropriate approach. This has to be done in a timely fashion to avoid any further treatments delay.

In most cases after returning from abroad, patients will be able to continue their treatment schedule immediately following their quarantine, however, there are certain scenarios where patients face delays. These include circumstances where protocols commenced abroad are either not internationally approved, individual medications are not Food and Drug Administration (FDA) or European Medicines Agency (EMA) approved, or medications are not readily available in the local hospital and/or the UAE. In those circumstances it is essential that insurance approval be obtained through early communication to insurance companies with treating physicians providing scientific evidence that supports the use of such protocol. More importantly, pharmacy management should be informed as early as possible about such medications so they can start the procurement process early, specifically when such medications are non-formulary and are not usually stocked in the hospital and/or the UAE.

In all cases, it is highly recommended that patients and their families understand the importance of communicating their treatment plan with their local hospital early, ideally during their quarantine period. This will help to expedite both the insurance pre-approval and the medication procurement process. Patients and their families also need to be aware that there are rare occasions where resuming the same protocol would not be possible, especially when scientific evidence is lacking and/or there is great concern for the safety of such protocol. In such circumstances it is essential that treating physicians have an open and clear communication with patients and their families on their first visit. Consequently, patients will understand what alternative options exist which are evidenced-based and readily available in the country. Moreover, this will save the patients’ time and minimize delays in starting treatment. Patients and their families should be informed about the risks associated with treatment delays, which will definitely compromise the patients’ clinical condition and oncological outcome. This is important as some patients may elect to hold their cancer treatment until the lockdown has been lifted and they are able to travel to resume their cancer care abroad.

Patient expectations from treatment outcome can be misunderstood or poorly communicated to the patients while being treated abroad due to either fixed beliefs about their conditions (e.g. denial) or inadequate translation during the clinic visits [9]. Therefore, establishing the treatment intent must be clearly discussed in the initial treatment evaluation.

There are many patients who were enrolled in clinical trials abroad who have halted their treatment and returned to the UAE. These patients face unique challenges as usually the same clinical trials are not available in the UAE and the experimental agents they are on may be unapproved and/or unavailable. In these circumstances we recommend that the treating physician recommends alternative treatment options after discussions with the patient, including explaining the practicalities related to clinical trial continuation in the UAE. Such interruptions ultimately affect patients who are currently on clinical trials as well as those who are not yet enrolled due to the temporary hold on clinical trials [12].

### Hematology

Hematological malignancies are acute, requiring urgent emergency care and, in most situations, the treatment has the potential to cure and therefore cannot be safely delayed as these cancers are likely more lethal than COVID-19 [13]. Despite the burden on the healthcare care systems created by COVID-19, this group of severely immunocompromised patients must be kept segregated in a sterile facility with no to minimal risk of contamination or infection. Unfortunately, this is becoming extremely difficult given the constraints on resources – both in terms of infrastructure and staffing. Ideally, there should be a dedicated facility to manage the full spectrum of hematological disorders (both malignant and non-malignant) with dedicated HCWs managing such patients to avoid any risk of cross contamination or posing a threat to this susceptible group.

With SARS-CoV2 infection, the pandemic is pressing the hematologists to take innovative steps that are deemed safer for patients. This includes facing challenges to keep the disease in remission and/or offer a line of therapy which bridges the gap between induction and more definitive treatment such as hematopoietic stem cell transplantation [14]. The use of 6 cycles of induction therapy has been recommended by the European Bone Marrow Transplant (EBMT) society, instead of 4, and to postpone the transplant until first relapse in standard-risk patients with multiple myeloma [15]. Patients with high-risk cytogenetics (with17p deletion) should still receive an induction, followed by high dose melphalan with ASCT. The use of lenalidomide maintenance in multiple myeloma can offer a safe therapy and continue to keep the remission, after a standard of care after induction treatment for multiple myeloma; clearly what is different in this equation is taking the autologous stem cell transplant out due to severe risks of immunosuppression over a long period.

Hypomethylating agents to hold the disease process in AML is another strategy in standard-risk disease and elderly patients. High risk or refractory AML has to be considered as an exception although these management decisions are to be made on a case by case basis.

Considering oral treatments to be taken at home; e.g.: Ibrutinib for patients with CLL rather than highly immunosuppressive hospital-based treatments is favored for response initiation [15].

The EBMT society has set clear guidelines for bone marrow transplant units during this pandemic. Although as the situation is variable between different geographic locations, it is also realized that the patients might suffer unnecessary harm if the transplant procedure or related cellular therapy is delayed. Adhering to local and national procedures and protocols is deemed essential to make an informed decision. The high-risk diseases, including acute myeloid leukemia, either presenting as high risk or refractory disease will need to proceed with allogeneic transplantation under strict precautions. All patients should be tested for SARS-CoV-2 and the test results should be negative before the start of the conditioning regardless of whether upper respiratory symptoms are present. In low risk patients, a deferral of 3 months has been recommended [15]. The difficulty in decision making comes for patients who are tested positive and the guidance is to defer the transplant conditioning to 14–21 days after 2 negative SARS-CoV-2 test. The cellular therapy with CAR-T for patients who are refractory, have to be given special individual considerations on a case by case basis. The guidelines have also been set for donors, staff and bone marrow transplant units [15].

The biggest challenge within the UAE is that there is no center for hematopoietic stem cell transplantation, offering autologous or allogeneic transplantation and, unfortunately, no post-transplant care including donor lymphocyte infusion for mixed chimerism [16–18], Extra-Corporeal Photopheresis for post-transplant chronic graft versus host disease [19, 20]. Hematopoietic stem cell transplant remains the standard of care for a variety of hematological malignancies, particularly multiple myeloma [21] and relapsed lymphoma [22]. Another dilemma facing patients returning to UAE is that after such intensive treatments, they have to be on strict monitoring for certain viruses (EBV, CMV) and immunosuppressive drug levels which, as are the tests mostly outsourced, ensue further delays in getting the results in days-weeks rather than a few hours as per standards. A state of art molecular laboratory service has become the need of the hour, as current pandemic restrictions mean that most diagnostic and molecular monitoring samples cannot be sent to outsourced services. Unfortunately, the knock-on effect is that many crucial investigations for both diagnosis and monitoring disease response are now not available.

Given the current pandemic scenario, the rational approach is to take measures to keep patients in remission, especially the relapsed lymphoma who might otherwise proceed to stem cell transplantation. The challenge to the physicians in this situation is to weigh the risks and benefits of delaying a standard therapy, or to continue extended consolidation/maintenance therapy with an accepted risk of treatment related toxicity to the patient. This also raises certain ethical concerns as to whom and when to treat [23].

As with most hematological cancers the goal of therapy is to achieve molecular remission, and targeted therapy has revolutionized the outcome for these patients. The majority of targeted therapy agents are biological agents with a significant level of financial burden [24]. The cost implications are not insignificant despite the availability of drugs. Unfortunately, the cost of these medications within the Middle East is also variable with a clear disparity between different Arab countries.

The parallel challenge is availability of blood and blood products and, during the current pandemic, there is a great reduction in the transfusion support products mainly due to decreases in donations [25].. This is largely due to the implementation of social distancing, cancellation of blood drives and low donor turnout owing to fears surrounding COVID-19. This issue has been observed globally and to address it the local bodies and governments have to increase the public awareness and build confidence to ensure safety of the donation process and the protocols being adopted by blood banks and hospitals [1, 26, 27]. The UAE is facing blood shortage and multiple public appeals have been made to support the blood transfusion services in the country [28] and the general population has been mobilized to make donations [29], for example, encouraging hospital staff to donate, especially in the absence of blood drives and donation services. The UAE Central Blood Bank releases the blood product ensuring there has been a replacement unit of donation provided by the treating hospitals and patients relatives. The cancellations of elective surgeries and implementing public awareness has helped overcome the shortage of blood products.

### Surgical oncology

While the management of cancer patients is based on a multimodality approach, surgical resection in many subtypes of cancer is the cornerstone for successful disease-free survival. Patients from the UAE seeking treatment for their respective oncological diseases were repatriated from different parts of the world with the intention of protecting Emirati patients during the COVID-19 pandemic. From the patients’ perspective, this would have occurred at different milestones of their surgical treatment journey including preoperative, immediate postoperative and recovery phase.

The key challenge faced by patients during the preoperative phase would mainly be a delay in surgical treatment. It is a common phenomenon that cancer patients in the UAE generally tend to present when symptomatic and often in the later stages of their illness.

Another preoperative challenge is the lengthening of the duration between neoadjuvant treatment and the surgical intervention as would be the case in breast, esophageal and lung cancers where patients would either receive a neoadjuvant regimen of either chemotherapy, radiotherapy, or both to be followed by surgical intervention [30–33]. The timing of surgery after the initial intervention is usually optimized to maximize the pathological response with careful considerations of risks vs benefits.

Postoperative challenges can be divided into initial postoperative (< 30 days) and recovery phases (> 30 days). Patients in the initial postoperative phase recovering at home after discharge are at risk for developing immediate postoperative complications such as anastomotic leaks, pneumonia, and failure to thrive due to issues with enteric feeding. Patients being repatriated immediately after surgery would have to deal with these issues in addition to the psychological stress brought on by the idea of traveling and risking the need for medical attention in air. Furthermore, travelling is compounded by the high deep venous thrombosis risk due to (1) active malignancy, (2) recent surgical procedure and (3) the immobility associated with prolonged aircraft travel for the respective number of hours needed to travel to the UAE. Kuipers et al. have shown that the risk of developing a venous thromboembolism increases 20 times in patients with recent surgery and 18 times in patients with an active diagnosis of cancer [34].

Finally, patients recovering from oncological surgery are at increased risk of complications from their perspective surgery that might require the intervention of local surgical oncology expertise. Such interventions would range from diagnosis of complications to consultation with ancillary services such as advanced endoscopy or interventional radiology, or even surgical re-intervention to resolve a complication. It is therefore important for patients being repatriated to identify a surgical oncologist in the respective disease field, prior to their return, to familiarize themselves with the patient’s disease and what has been done thus far to be able to resume the treatment process and to monitor the patient for recurrence of disease.

### Radiation

Radiation Therapy (RT) is a critical step in cancer treatment that is used in 50–60% of all patients as part of their treatment [35]. In addition to cancer being a risk factor for potential increased morbidity and mortality related to COVID-19 infection, RT is often offered to elderly, surgically-non-fit patients who are also at a higher risk of infection complications [36]. Therefore, cancer patients receiving RT might be one of the most vulnerable group of patients which requires more careful consideration amidst the pandemic.

Whether offered as part of patient’s definitive/curative or palliative treatment intent, RT is most commonly given as once daily treatment over a pre-specified treatment course designated by the patient’s primary treating radiation oncologist. Several institutional guidelines are being implemented across major cancer centers in the world where many cancer patients from the UAE are receiving their treatment [37]. Moreover, national and international radiation oncology societies such as The American Society of Radiation Oncology (ASTRO), European Society for Radiotherapy and Oncology (ESTRO), and the Global Radiation Oncology Targeted Response have issued practice RT guidelines during the pandemic [38]. These resources, as well as other site-specific guidelines and consensus provides an excellent general framework for RT practice [39–41]. However, a gap remains for international patients receiving their treatment abroad who have been asked or are contemplating a return to their home country.

It is well established that radiation treatment interruptions should be minimized as it is associated with increased risk of treatment failure [42]. This is especially important in the setting where RT is used as a definitive treatment modality for diseases such as advanced head and neck cancers as well as in certain gynecological malignancies and other cancers [42, 43]. If treatment interruptions happen, especially if prolonged, RT dose-compensation calculation should account for all known radiobiological factors involved. Yet these calculations are based on radiobiological and mathematical models and do not substitute the value of uninterrupted treatment [44]. Therefore, patients actively undergoing definitive radiation treatment are usually advised against treatment interruptions and breaks unless clinically warranted.

On the other hand, for patients who have not started their treatment yet, or those who do not require immediate RT planning and administration as per current guidelines [38], and given the uncertainty that continues in the current pandemic and the potential future travel limitations that may happen, dynamic arrangement and scheduling should be considered. The first critical step in doing so is communication. Institutions having international patients who are requested or seek to return home should establish methods of virtual communication and secure data sharing with local radiation oncology facilities in the patients’ respective countries. By doing this, each individual case can be discussed in detail. Moreover, the proposed RT treatment plan can be designed specifically for each case, depending on the local radiation oncology facility expertise, available treatment modalities such as Intensity Modulated Radiotherapy (IMRT), Volumetric Modulated Arc Radiotherapy (VMAT), Stereotactic Body Radiotherapy and Radiosurgery (SBRT/SRS), and lastly the type of Image Guided Radiotherapy (IGRT) availability. By establishing secure data sharing platforms between local and international institutions, RT plans can be shared and reviewed amongst both radiation oncology treatment teams providing an additional quality assurance and peer-to-peer review step. Furthermore, the patient’s RT plan can be saved at both facilities, which might be essential for current and future plan adjustments or re-treatments that the patients might require. For patients who are receiving RT as part of a research protocol, we believe that they should continue to be treated as per protocol if safe and feasible. Moreover, the communication between local and international facility can ensure if protocol RT requirement and dose constraints are met.

Finally, for patients who are finishing their RT abroad and returning soon, or for those who finished their treatment abroad months ago and were planning to travel again for their upcoming follow up appointments, communication between patient’s local and the radiation oncologist abroad is also vital. This allows radiation oncologists to discuss the patient’s RT treatment plan and course in detail and to review all early RT-related side effects encountered during treatment. Thus, helping in monitoring and predicting potential late RT-related side effects, and arranging subsequent local follow up appointments accordingly.

### Radiology and imaging

It is known that radiology plays an integral role in oncology care which include screening, establishment of a diagnosis, staging and minimally invasive intervention. To reach a diagnosis after interpreting images, radiologists need to be provided with clinical history of the patient’s status and treatment received, if applicable. Radiologists are already in collaboration with medical and surgical oncologists as well as radiation oncologists and are vital members of the MDT involved in the treatment of oncology patients. Disease staging using conventional x-rays, CT scans, MRI, PET scan and nuclear studies will determine the course of treatment that is needed and is the primary determinant of prognosis [45]. Follow-up radiological studies are essential to assess a state of remission and/or check for recurrence. Therefore, physicians, particularly, oncologists should understand the contribution and limitations of radiology and be aware of the new constantly developing techniques to improve patient care emerging in the years ahead [45].

With the current COVID-19 pandemic, radiologists in the UAE are facing a unique, yet, challenging situation. We will describe the preparedness of radiology departments and the radiologist’s role in the management of oncology patients and the challenges encountered in the current time of COVID-19 in the UAE, especially with returning cancer patients.

Most radiology departments in coordination with local regulatory bodies took the decision to postpone and reschedule all elective and non-urgent imaging cases. In addition, all in-patients requiring a radiological study are required to have a Reverse transcription polymerase chain reaction (RT-PCR) test to exclude COVID-19 prior to any imaging studies. In patients who test positive for COVID-19 but require urgent imaging, higher levels of infection control is implemented to minimize exposure to HCWs and other patients.

Returning cancer patients represent a challenge for the treating oncologist, given that they were at different treatment stages and had different radiological studies at baseline, staging and follow up. Unfortunately, most of them were unable to obtain reports and soft copies of their previous radiological studies which led to treatment delay and interruption. The hurdle radiologists face with reimaging is that they lack previous radiological studies for comparison and can only report the current images findings without being able to correlate with previous images, which is critical in cancer treatments decision. To overcome this problem and, as discussed earlier, local oncologists may contact treating oncologists abroad requesting a copy of the radiological studies. In case a hard copy is being provided, courier services will be an issue because of flight restrictions, therefore, in our opinion, agreements with international cancer centers in having a secure cloud sharing service to browse patients’ records is recommended to avoid unnecessary reimaging and treatment interruption and minimize the patient’s treatment delay. We still also recommend obtaining a baseline imaging for newly returned patients if previous images are not available to serve as a new baseline images for future comparison.

### Pathology

Optimal cancer care begins with an accurate diagnosis. Patients returning from cancer care abroad will have previously undergone pathological valuation. Their prescribed oncology treatment will be based on the histologic classification, pathologic stage and possibly genomic analysis of the tumour. For these patients, it may be useful to review the pathology report in order to assess the appropriateness of the prescribed treatment regimen. Most cancer centres will discharge patients with a copy of their medical records and it is a matter of requesting documentation and, if feasible, tissue blocks or slides. At a minimum, the pathology report should be reviewed by an in-house pathologist. The accuracy of the diagnosis can often be assessed by correlating the clinical findings, histologic description, and relevance of the immunohistochemical workup and interpretation. Ideally, the diagnosis should be confirmed by histologic examination of the tumour, achieved by review of the submitted slides or recuts of the tissue blocks. The tissue blocks can be useful later for additional work-up for prognostic or predictive markers, genomic studies, or comparison with metastasis. If the slides or tissue blocks are not available, the pathologist can communicate with the referring institution and request digital images of the tumour. Final confirmation of the diagnosis should be rendered in correlation with the clinical and surgical history, best accomplished by comprehensive review in an MDT setting.

Patients returning from abroad without a confirmed diagnosis present a more challenging situation. Collaboration between the various members of the MDT is required to integrate all available clinical and surgical data, imaging studies, and pathology workup. If there remains no consensus diagnosis, a biopsy should be obtained. The risk of biopsy must be weighed against the relative benefits of obtaining a definitive classification of the tumour.

Of equal importance in both of these patient groups is the need to minimize the risk of acquiring infection by exposure to staff and other patients while visiting the hospital. Although there are currently no reports regarding a higher incidence of COVID-19 asymptomatic infections in patients with cancer, there is data to suggest a higher risk of morbidity and mortality [46]. RT-PCR SARS-CoV-2 testing should be proposed to all patients undergoing surgery, radiotherapy, chemotherapy or immunotherapy. If feasible, antibody tests should also be performed to identify previous COVID-19 infection in all cancer patients [47].

Of note in cases of oncologic surgery requiring frozen section: there should be communication between the surgeon and pathology department to identify suspected/confirmed patients prior to the frozen section procedure. The surgeon and pathologist should discuss the value of the frozen section and the possibility of an alternative course of action. Frozen section should be considered for patients where it can reasonably be expected to give an accurate and actionable diagnosis. The Lab should be advised to follow a contagion protocol similar to that for patients suspected of having tuberculosis [48].

### Palliative and supportive care

The aim of palliative care is to improve the quality of life of a patient diagnosed with cancer as they progress through their cancer treatment pathway. The seminal paper by Jennifer Temel in 2010 [49], demonstrated that early integration of palliative care into the oncology treatment pathway for patients with advanced lung cancer resulted in improved symptom control and quality of life measures compared to patients who only received palliative care later in their treatment pathway. This supportive approach of palliative care alongside oncology treatment is now enshrined in the ASCO clinical guidelines [50]. Patients receiving oncology treatment abroad will often have received some palliative care input. On their return to the UAE, a number of issues are likely to arise. Patients who have received palliative care input abroad for symptom control usually return to the UAE with a small supply of medication, including opioid analgesics, anti-emetics, neuropathic pain medicines and sedatives. On their arrival in the UAE, patients will be required to either try and source these medications (if registered with the local health authority) or to source an alternative, which carries the risk of upsetting finely adjusted symptom control. While the UAE has increased the range of medications available to patients including controlled medicines, access to these medications remains very tightly regulated with restrictive limits on who is able to prescribe and the maximum prescribed amount [51]. Patients have often received input from multiple members of the palliative care team including physicians, specialist nurses, social workers, psychologists and chaplains. Without comprehensive documentation and handover, the insights gained into how best to support the patient and their family is lost. This includes conversations about patient and family fears, preferences for treatment, goals of care and advanced care planning. This is particularly pertinent in cases where patients have very advanced metastatic disease and much effort has gone into discussing what treatment options are appropriate and why and how they can be supported and cared for when/if they are no longer well enough for active anti-cancer treatment. This is best reflected in cases where such a patient has a DNAR (Do Not Attempt Resuscitation) status whilst abroad but, in most federal regions UAE, there is no equivalent status. While the Federal Decree Law on Medical Liability (Law No. 4 of 2016) allows for natural death by refraining from performing cardiopulmonary resuscitation (CPR) on terminally ill or dying patients, unlike a DNAR status, cardiopulmonary resuscitation cannot be denied if the patient expressly requests it, even if resuscitation is likely to be futile. This inconsistency can increase patient and family distress and precipitate a degree of moral distress among the HCWs who feel that it is inappropriate to subject a patient to an aggressive medical intervention when it is clear that the intervention will not be successful in reversing the dying process [52]. When feasible, a comprehensive clinical handover, including any palliative care input, is essential for optimal patient care to ensure consistent messaging and prevent any expectation mismatch among patient and family members.

### Post pandemic outflux

It is expected that once the travel restrictions and the lockdown status in the UAE are lifted, a significant number of patients will resume their medical tourism abroad. This should be expected and planned for accordingly. Patients and their families should be counseled about the risk of interruption of their treatment plan which may have a detrimental effect on their oncological outcomes. Patients should also be counseled about the uncertainties regarding the status of the countries they are traveling to, since each country will have their own rules and regulations regarding travel, quarantine and accessibility to health care services based on their available resources. If patients prefer to travel, then clear documentations and communication with the receiving health facility abroad should be established as early as possible and patients should be provided with all required information and items (medical reports, images, pathology slides and blocks) to facilitate a safe transfer of care. Early planned discussion is recommended to understand and anticipate patients’ wishes to continue treatment in UAE or to travel abroad again once the lockdown is lifted and, accordingly, is very critical to plan treatment.

## Conclusion

In this review we discussed some of the practical challenges and recommendations for cancer patients returning to the UAE from treatment abroad. This may be a temporary issue, yet it is critical to have a consensus in the best approach to address such a vulnerable population and to provide guidance during this challenging pandemic. The importance of a thorough clinical handover cannot be overstated and regulatory bodies are needed to prevent what can be considered unethical procedure towards returning cancer patients. Clear communication is paramount to gain the trust of returning patients and their families. Trust will ensure that patients and their families are able to develop a good rapport with the treating oncology team, which will facilitate smoother discussions of treatment options and continuation of their cancer treatment in the UAE. This pandemic may also serve as an opportunity to encourage patients to receive treatment locally. Future studies are recommended to address the steps needed to retain cancer patients to undergo cancer care in the UAE, rather than seeking treatment abroad.

## Data Availability

Not applicable.

## Abbreviations

ASTRO: American Society of Radiation Oncology
COVID-19: Coronavirus disease 2019
CMV: Cytomegalovirus
CPR: Cardiopulmonary resuscitation
DNAR: Do Not Attempt Resuscitation
EBMT: European Bone Marrow Transplant
EBV: Epstein-Barr virus
EMA: European Medicines Agency
ESTRO: European Society for Radiotherapy and Oncology
FDA: Food and Drug Administration
HCWs: Health care workers
IGRT: Image Guided Radiotherapy
IMRT: Intensity Modulated Radiotherapy
MDT: Multidisciplinary team
RT: Radiotherapy
PCR: Polymerase Chain Reaction
RT-PCR: Reverse transcription polymerase chain reaction
SARS-CoV-2: Severe Acute Respiratory Syndrome Coronavirus 2
SBRT/SRS: Stereotactic Body Radiotherapy and Radiosurgery
UAE: United Arab Emirates
VMAT: Volumetric Modulated Arc Radiotherapy
